# Effects of dietary *n*-6: *n*-3 polyunsaturated fatty acid ratios on meat quality, carcass characteristics, tissue fatty acid profiles, and expression of lipogenic genes in growing goats

**DOI:** 10.1371/journal.pone.0188369

**Published:** 2018-08-01

**Authors:** Mahdi Ebrahimi, Mohamed Ali Rajion, Saeid Jafari, Mohammad Faseleh Jahromi, Ehsan Oskoueian, Awis Qurni Sazili, Yong Meng Goh, Morteza Hosseini Ghaffari

**Affiliations:** 1 Department of Veterinary Preclinical Sciences, Faculty of Veterinary Medicine, Universiti Putra Malaysia, Serdang, Malaysia; 2 Department of Plant Sciences & Biotechnology, Faculty of Life Sciences & Biotechnology, Shahid Beheshti University, Tehran, Iran; 3 Department of Poultry Science, Faculty of Agriculture, Tarbiat Modares University, Tehran, Iran; 4 Institute of Tropical Agriculture, University Putra Malaysia, Serdang, Malaysia; 5 Department of Animal Sciences, Faculty of Agriculture, University Putra Malaysia, Serdang, Malaysia; 6 Department of Agricultural, Food and Nutritional Science, University of Alberta, Edmonton, Canada; Wageningen UR Livestock Research, NETHERLANDS

## Abstract

The present study was conducted to investigate the effects of altering the ratio of *n*-6 to *n*-3 fatty acids in the diet on meat quality, fatty acid composition of muscle, and expression of lipogenic genes in the muscle of Boer goats. A total of twenty-one Boer goats (5 months old; 31.66±1.07 kg body weight) were randomly assigned to three dietary treatments with *n*-6:*n*-3 fatty acid ratios of 2.27:1 (LR), 5.01:1 (MR) and 10.38:1 (HR), fed at 3.7% of body weight. After 100 days of feeding, all goats were slaughtered and the *longissimus dorsi* muscle was sampled for analysis of fatty acids and gene expression. The dietary treatments did not affect (*P*>0.05) the carcass traits, and meat quality of growing goats. The concentrations of *cis*-9,*trans*-11 conjugated linoleic acid, trans vaccenic acid, polyunsaturated fatty acids, and unsaturated/saturated fatty acid ratios linearly increased (*P*<0.01) with decreasing dietary *n*-6:*n*-3 fatty acid ratios, especially for LR in the *longissimus dorsi* muscle of goats. In contrast, the mRNA expression level of the PPARα and PPARγ was down-regulated and stearoyl-CoA desaturase up-regulated in the *longissimus dorsi* of growing goats with increasing dietary *n*-6:*n*-3 fatty acid ratios (*P*<0.01). In conclusion, the results obtained indicate that the optimal *n*-6:*n*-3 fatty acid ratio of 2.27:1 exerted beneficial effects on meat fatty acid profiles, leading towards an enrichment in *n*-3 polyunsaturated fatty acids and conjugated linoleic acid in goat intramuscular fat.

## Introduction

Over the last decade, there has been growing efforts in producing healthier meat, with a more favorable balance of n-3 versus n-6 polyunsaturated fatty acids (PUFA; lower *n*-6:*n*-3 ratio) and higher conjugated linoleic acid (CLA) content. The ratio of *n*-6 to *n*-3 FA in meat may be more important than the levels of *n*-3 PUFA intake for decreasing the risk of cardiovascular disease and other chronic disorders [1].

The *n*-6 and *n*-3 PUFA ratio is highly influenced by the fatty acid (FA) composition of the diet fed to the ruminants and affects the concentration of CLA in the rumen, milk, and meat [2]. The CLA acronym refers to a group of positional and geometric isomers of C18:2*n*-6 (linoleic acid, LA), and many studies suggest that CLA exhibits anti-carcinogenic, anti-adipogenic, anti-diabetogenic, anti-atherogenic and anti-inflammatory properties [3, 4]. Ruminant fats are among the richest natural sources of CLA isomers, particularly of *cis*-9, *trans*-11 CLA, and are the main sources of these isomers in the human diet [4]. The *cis*-9, *trans*-11CLA is produced during ruminal biohydrogenation (BH) of LA to stearic acid and by endogenous conversion of C18:1 *trans*-11 by Δ9-desaturase in tissues [5, 6].

The PUFA are one of the best endogenous or natural activators of PPAR [7, 8]. *In vivo* and *in vitro* studies have shown that PPARα is activated by FAs [9]. For example, mutagenesis of the PPARα gene by homologous recombination causes resistance to the induction of the enzymes of the peroxisomal β-oxidation pathway by peroxisome proliferators [10]. In most cases, these studies have been carried out in the mouse, where lipid metabolism is different from that in ruminants. In addition, these studies have taken into account only the effects of *n*-6:*n-*3 PUFA ratio, without considering the possible effects of the other FAs on gene expression.

Although many studies have been published describing manipulation of the FA composition of animal meat [11, 12, 13], less attention was given to the ratio of n-6 to n-3 FA in meat. Therefore, the present study was conducted to determine the effect of modifying the dietary *n*-6:*n*-3 PUFA ratio on growth performance, carcass characteristics, meat quality, FA composition of the *longissimus dorsi* (LD) muscle, antioxidant activity and PPARα, PPARγ and Stearoyl-CoA desaturase (SCD) gene expression in LD muscle of Boer goats.

## Materials and methods

### Experimental plant materials and their chemical analyses

Fresh oil palm fronds (OPF) used in this experiment were harvested in the fields of the Malaysian Agricultural Research and Development Institute (MARDI) located in Serdang, Selangor, Malaysia (3°00′18.88″N, 101°42′15.05″E). The OPF samples were oven dried at 55 ^o^C for 2 days, and stored at -80 ^o^C for further analysis and use in the animal feeding trial. The dry matter (DM) content and crude protein (CP, total nitrogen × 6.25) were determined using method number 934.1 and 990.03 of the AOAC [14] respectively. The DM was determined by drying 10 g of fresh samples at 60°C in a forced air oven for 48 h. Neutral detergent fiber (NDF) and acid detergent fiber (ADF) were determined using heat-stable amylase and sodium sulphite, according to Van Soest et al [15]. Values for NDF and ADF were expressed as inclusive of residual ash.

### Animal welfare

This study was conducted at Universiti Putra Malaysia under the guidelines of the Research Policy of the Universiti Putra Malaysia on Animal welfare and ethics. The experimental protocol was approved by the Universiti Putra Malaysia Animal use and care committee. The care of the experimental goats was in accordance to Malaysian standards.

### Animals, diets, and management

Twenty-one male Boer goats (Five-month-old; 13.66 ± 1.07 kg of body weight, BW) were used in a completely randomized design in which animals were randomly assigned to 1 of the following 3 diets differing in *n*-6:*n*-3 PUFA ratios:2.27:1 (LR), 5.01:1 (MR), and 10.38:1 (HR). Goats were initially drenched against parasites and housed individually in wooden pens measuring 1.2 m×1 m each, built inside a shed with slatted floor 0.5 meters above ground. We traced animals using history of individuals; the males’ goats were not relative. The feed ingredients and composition of experimental diets are shown in Table 1. Oleic and LA were the predominant FA in all the treatment diets, whereas the LR diet showed a more balanced supply of the three main FA of plant origin namely oleic, LA and LNA although the latter was quantitatively the most important (Table 1). The percentage of FA contents of oil sources used to adjust the *n*-6:*n-3 PUFA* ratio is shown in Table 2. The linseed (LO) was used as a source of LNA while sunflower oil (SO) was used as a source of LA. The experimental diets were fed daily at 3.7% of BW (DM basis; Jurgens [16]), with adjustments made weekly according to the changing BW. The concentrate (70% DM basis) and OPF silage (30% DM basis) were mixed and offered in 2 equal meals at 0800 am and 0500 pm. The diets were formulated to be isonitrogenous and isocaloric and to meet the energy and protein requirements of growing goats as described by NRC [17]. All goats had free access to water and mineral block. The feeding trial lasted for 100 days with a three-week adaptation period.

**Table 1:** Ingredients and chemical composition of the experimental diets.

Item	Dietary *n*-6:*n*-3 PUFA ratio ^1^		
	LR	MR	HR
**Ingredient(as-fed)**			
** OPF silage (%)**	30.00	30.00	30.00
** Corn, grain (%)**	17.00	17.00	17.00
** Soybean meal (%)**	13.30	13.30	13.30
** Palm kernel cake (%)**	25.11	25.11	25.11
** Rice bran (%)**	8.18	8.18	8.18
** Linseed oil (%)**	1.30	0.40	0.00
** Palm kernel oil (%)**	0.10	1.00	1.10
** Sunflower oil (%)**	2.00	2.00	2.30
** Mineral premix (%)**	0.50	0.50	0.50
** Vitamin premix (%)**	0.50	0.50	0.50
** Ammonium chloride (%)**	1.00	1.00	1.00
** Limestone (%)**	1.00	1.00	1.00
**Chemical composition**			
** ME (Mcal/Kg)**	2.51	2.51	2.51
** CP (%)**	13.00	13.00	13.00
** EE (%)**	7.00	7.00	7.00
** NDF (%)**	48.90	48.90	48.90
** ADF (%)**	33.00	33.00	33.00
** Ca (%)**	0.68	0.68	0.68
** P (%)**	0.36	0.36	0.36
**Fatty acid composition (% of total identified FAs)**			
** SFA** ^**2**^	27.18	32.99	32.97
** MUFA**^**3**^	20.45	27.99	27.86
** n-3 PUFA**^**4**^	13.63	6.47	3.44
** n-6 PUFA**^**5**^	30.92	32.40	35.68
** n-6: n-3 FAR**^**6**^	2.27	5.01	10.38

^1^LR: low *n*-6:*n*-3PUFA ratio (2.27:1); MR: medium *n*-6:*n*-3PUFA ratio (5.01:1); HR: high *n*-6:*n*-3PUFA ratio (10.38:1).

^2^SFA = (C10:0 + C12:0 + C14:0 + C16:0 + C17:0 + C18:0)

^3^Total monounsaturated FA (MUFA, C16:1 + C18:1n-9)

^4^ n-3 PUFA = (C18:3n-3)

^5^ n-6 PUFA = (C18:2n-6)

^6^*n*-6:*n*-3 FAR = (C18:2n-6)/ (C18:3n-3).

**Table 2:** The fatty acid (FA) contents (% of total identified FAs) of oil sources used to adjust the *n*-6:*n*-3FA ratios.

Oil source	C14:0	C16:0	C18:0	C18:1n-9	C18:2n-6	C18:3n-3
***SFO***^***1***^	-	6.40	3.66	28.32	61.23	0.39
***PKO***^***2***^	16.75	9	2.36	16.62	18.47	-
***LO***^***3***^	-	5.15	3.17	16.62	16.12	57.09

^1^SFO: sunflower oil (Lam Soon Edible Oils Sdn. Bhd.)

^2^PKO: palm kernel oil (Malaysian Palm Oil Board)

^3^LO: Linseed oil (Brenntag Canada, Inc.), C14:0, myristic acid; C16:0, palmitic acid; C18:0, stearic acid (SA); C18:1, oleic acid; C18:2n-6, linoleic acid (LA); C18:3n-3, linolenic acid (LNA).

### Slaughter procedures and measurement of carcass characteristics

At the end of the 100-day trial, all the animals were slaughtered by cutting throat accordance with the standard slaughter procedures outlined in the MS 1500:2004 (Department of Standards Malaysia) at the Meat Science laboratory, Department of Animal Science, Faculty of Agriculture, Universiti Putra Malaysia. Approximately 150 g of the LD muscle from 12^th^ to 15^th^ rib were removed from the carcass. All samples were stored at -80°C until analyzed for long chain FA, meat quality, and antioxidant activity. After 24 h, the chilled carcass weight was noted then the carcasses were split into equal halves along the midline using a carcass saw. The right half was dissected immediately for muscle sampling. The right half carcass was ribbed between the 12^th^ and 13^th^ rib. The corresponding back fat thickness was determined from this cut using an aluminum ruler. The rib eye area was determined using an Image J software package (National Institutes of Health, Bethesda, MD, USA).

The dissection technique used for measuring muscle, bone, and fat composition was described by Colomer-Rocher et al [18]. The right portion of each carcass was weighed and then divided into five primal cuts namely the neck, shoulder, breast, loin and leg and the cuts were weighed and expressed as a percentage of the total weight of the carcass. Each cut was dissected into components of meat, bone, subcutaneous fat, and intermuscular fat. Firstly, the muscle was individually removed from their attachment and then the external fat was removed. Muscle, fat, and bone were separated and weighed individually.

### Determination of meat quality

The pH of meat samples was measured at 1, 3 and 6 days post- mortem by inserting a pH electrode connected to a portable pH meter (Hanna Instruments, Woonsocket, RI, USA) set to record at 5°C.

Instrumental color was measured using a ColorFlex system (Hunter Associates Laboratory, Reston, USA). The samples were evaluated for lightness (L*), redness (a*), yellowness (b*), hue angle (arctan, b*/a*), which describes the hue or color of the meat and saturation index or chroma calculated as √(*a*^2^ + *b*^2^) which describes the brightness or vividness of color [19]. All values were determined from the mean of seven measurements of each muscle at 28 ± 2°C using the 10˚ standard observer. The spectrocolorimeter was standardized using white (L* = 100), and black (L* = 0) standard tiles, before being used. Samples were allowed to thaw at 4°C overnight prior to analysis. The samples were placed directly into the color meter and measured. Three readings of the L*, a* and b* values and spectral reflectance (400–700 nm) were collected from different sites of each sample and averaged.

To determine drip loss, samples of approximately 30 g taken 24 h, 3 days and 6 days post-slaughter were trimmed and weighed. Each sample was then placed in a square netting (25 cm^2^) and suspended in an inflated plastic bag for 48 h at 4°C, without making any contact with the bag, after which they were re-weighed [19]. Drip loss was calculated as the percentage weight change [19].

Driploss(%)=[(W1−W2)/W1]×100

Where; W1 is the initial weight, W2 is the weight of the raw sample.

For determination of cooking loss, the meat samples were placed in polyethylene bags and inserted in a hot water bath (80°C) until the internal temperature (measured using a thermometer with a hand probe; Hanna Instruments, Woonsocket, RI, USA) reached 78°C. Goat meat samples in the bags were then cooled under tap water for 15 min. The cooled samples were taken from the polyethylene bag, blotted dry with paper towels and weighed. Cooking loss was estimated by weighing them before and after cooking [19].

$$Cooking\;loss\;(\%)=\left[\frac{W2-W3}{W2}\right]\times 100$$

Where; W2 is the weight of the raw sample and W3 is the final weight.

Warner–Bratzler shear force (WBS) measurement was performed on three cores (1.27 cm diameters) removed from the LD meat parallel to the longitudinal orientation of the muscle fibers. The WBS was determined using A. HD plus a texture analyzer (Stable Micro System, Surrey, UK) fitted with a Warner Bratzler blade device. Each core was placed on the base plate of the texture analyzer and sheared once in the center and perpendicular to the longitudinal direction of the fibers. Warner-Bratzler shear force values were reported as the mean of all core values of each individual sample. A lower shear force value (Newton, N) indicates more tender meat whereas a higher shear forces result indicating tougher meat.

Lipid oxidation of meat samples was measured using thiobarbituric acid-reactive substances (TBARS) according to the method of Lynch and Frei [20], modified by Mercier et al [21]. Meat samples (1 g) were homogenized in 4 mL of 0.15 M KCl + 0.1m*M* BHT with Ultraturrax (1 min, medium speed). After homogenization, 200 μL of the sample were mixed with TBRAS solution and then heated in a water bath at 95°C for 60 min until the development of a pink color. After cooling, 1 mL of distilled water and 3 mL of n-butyl alcohol were added to the extracts and vortexed. The mixtures were centrifuged at 5000 (rpm) for 10 min. The absorbance of the supernatant was read against an appropriate blank at 532 nm using a spectrophotometer (Secomam, Domont, France). The TBARS were calculated from a standard curve of 1, 1, 3, 3- tetraethoxypropane and expressed as mg malondialdehyde (MDA) /Kg sample.

### Fatty acid analyses of experimental diets and tissue *longissimus dorsi*

For FA analysis, total fat was extracted from the experimental samples and tissue using chloroform: methanol following the method described by Ebrahimi et al [22]. The extracted fat was saponified with ethanolic KOH for 10 min at 90°C. Fatty acids were converted to FA methyl esters (FAME) by transesterification with methanolic boron trifluoride (14% BF_3_) at 90°C for 20 min. The FAME were then analyzed by gas–liquid chromatography (Agilent 7890A). 1 μL of FAME was injected by an auto sampler into the chromatograph, equipped with a flame ionization detector (FID). The helium carrier gas was used at a flow rate of 1 mL × min^-1^. The FAME were separated on a 100 m × 0.32 mm × 0.25 μm film thickness using Supelco SP-2560 capillary column (Supelco, Inc., Bellefonte, PA, USA). The injector and detector temperature was maintained at 250°C and 300°C respectively. The column was operated isothermally at 120°C for 5 min, then programmed to 250°C at 4°C/min, increased by 2°C/min up to 170°C, held at 170°C for 15 min, increased again by 5°C/min up to 200°C, and held at 200°C for 5 min and then increased again by 2°C/min to a final temperature of 235°C and held for 10 min. Peak identification was performed by using known standards (mix C4–C24 methyl esters and CLA isomers; Sigma-Aldrich, Inc., St. Louis, Missouri, USA) and relative quantification was automatically carried out by peak integration.

### Quantification of tissue PPARs and SCD gene expressions by real-time polymerase chain reaction (PCR)

Immediately after slaughtering, the LD muscle was quickly excised and snap-frozen in liquid nitrogen and stored at -80°C until RNA extraction. Total RNA was extracted from 100 mg of frozen tissue using the RNeasy®lipid tissue mini kit (Cat. No. 74804, Qiagen, Hilden, Germany) and DNase digestion was completed during RNA purification using the RNase-Free DNase set (Qiagen, Hilden, Germany) according to the manufacturer’s instructions. Total RNA purity was determined by the 260/280 nm ratio of absorbance readings using NanoDrop ND-1000 UV-Vis Spectrophotometer (NanoDrop Technologies, Wilmington, DE, USA). Purified total RNA (1 μg) was reverse transcribed using a Quantitect^®^ reverse transcription kit (Qiagen, Hilden, Germany) in accordance with the manufacturer’s recommended procedure.

Real-time PCR was performed with the Bio-Rad CFX96 Touch (Bio-Rad Laboratories, Hercules, CA, USA) using optical grade plates using Quantifast^®^ SYBR green PCR kit (Cat. no. 204054, Qiagen, Hilden, Germany). The sequences of primers are shown in Table 3. β-actin [23], *PPARα*, *PPARγ* and SCD [24] were used in this study.

**Table 3:** Names and sequences of the primers used for gene expression study.

Target group		Sequence 5’– 3’	Length^3^	Reference
***β-actin***	F^1^	CGC CAT GGA TGA TGA TAT TGC	123	Waters et al [23]
	R^2^	AAG CGG CCT TGC ACA T		
***PPARα***	F	TGC CAA GAT CTG AAA AAG CA	101	Dervishi et al [24]
	R	CCT CTT GGC CAG AGA CTT GA		
***PPARγ***	F	CTT GCT GTG GGG ATG TCT C	121	Dervishi et al [24]
	R	GGT CAG CAG ACT CTG GGT TC		
***SCD***	F	CCC AGC TGT CAG AGA AAA GG		Dervishi et al [24]
	R	GAT GAA GCA CAA CAG CAG GA	115	

^1^F: forward

^2^R: reverse

^3^Length (nm)

The β-actin was used as the reference gene to normalize the tested genes. All primers were purchased through 1^st^ BASE oligonucleotide synthesis (1^st^ Base, Singapore). Each reaction (20 μl) contained 8.5 μL SYBR green PCR mix, 1 μL cDNA, 1 μL each of forward and reverse primers and 8.5 μL RNase-free water. Target genes were amplified through the following thermo cycling program: 95°C for 10´, 40 PCR cycles at 95°C for 30˝, 60°C for 20˝ and 72°C for 20˝. Fluorescence was measured at every 15˝ to construct the melting curve.

A real-time PCR was conducted for each primer pair in which cDNA samples were substituted with dH_2_O to verify that exogenous DNA was not present. Additionally, 1 μg of RNA isolated by the procedure described above was substituted for cDNA in a real-time PCR reaction to confirm that there were no genomic DNA contaminants in the RNA samples. Both negative controls showed no amplification after 40 cycles. The efficiency of amplification was determined for each primer pair using serial dilutions. The cycle numbers at which amplified DNA samples exceeded a computer generated fluorescence threshold level were normalized and compared to determine relative gene expression. Higher cycle number values indicated lower initial concentrations of cDNA and thus lower levels of mRNA expression. Each sample was run in triplicate, and averaged triplicates were used to assign cycle threshold (CT) values. The ΔCT values were generated by subtracting experimental CT values from the CT values for β-actin targets co-amplified with each sample. The group with the highest means ΔCT value (lowest gene expression) per amplified gene target was set to zero and the mean ΔCT values of the other groups were set relative to this calibrator (ΔΔCT). The ΔΔCT values were calculated as powers of 2 (-2ΔΔCT), to account for the exponential doubling of the PCR. Expression of PPARα, PPARγ and SCD in the LD muscle of treatment groups compared to HR group are shown in Tables 2, 3 and 4, respectively.

### Statistical analysis

Results were analyzed using analysis of variance with *n*-6:*n*-3 PUFA ratios as the main effect and initial weight as a covariate. The initial and hot carcass weights were used in the model as covariates because they had a significant effect on some variables. When covariance was not significant, it was removed from the model. Feed intake, ADG, and gain-to-feed ratio were analyzed using a repeated-measures mixed model (PROC MIXED) of SAS (SAS Inst. Inc., Cary, NC), including the goat as the random component of the model and diet (LR, MR, and HR), age (week), and their interaction as fixed components. The meat quality data was analyzed as a completely randomized design with a factorial experiment. A 3 × 3 factorial design (diets **×** postmortem periods) was employed for data (color, drip loss, cooking loss, shear force and TBARS values). Carcass characteristics, FA data, and all the gene expression data were analyzed by one-way ANOVA, using the MIXED procedure of the SAS. Some real-time PCR data which did not meet the ANOVA requirement of normality were log10-transformed for the analysis. Means were separated using the “pdiff” option of the “lsmeans” statement of the MIXED procedure. Differences of *P*<0.05 were considered to be significant. The data were checked for normality using PROC UNIVARIATE of SAS [25] software and the results in the tables are presented as means ± standard error of the mean.

## Results

### Carcass characteristics

The carcass characteristics and traits of goats fed diets with varying*n*-6:*n*-3 PUFA ratios are presented in Table 4.The dressing percentages of the goats decreased linearly (*P*<0.04) with increasing the dietary *n-6*:*n-3* PUFA ratios. The back fat thicknesses (P>0.05) decreased linearly as the dietary ratio of *n-6*:*n-3* PUFA increased. The rib eye muscle area decreased linearly (*P*<0.01) with the increase in the dietary ratio of *n*-6:*n-*3 PUFA. The warm and cold carcass weights as well as subcutaneous and intermuscular fat were not affected (*P*>0.05) by the different *n-6*:*n-3* PUFA ratios. The percentages of meat, bone, neck, shoulder, breast, loin and leg cuts were not affected (*P*>0.05) by the dietary ratio of *n-6*:*n-3* PUFA after 100 days of dietary treatment.

**Table 4:** Effect of dietary *n*-6:*n*-3 PUFA ratios on carcass characteristics and primal cuts of goats.

Item	Dietary *n*-6:*n*-3 PUFA ratios^a^			SEM	*P*-value	
	LR	MR	HR		Linear	Quadratic
**Dressing (%)**	45.06^a^	42.94^b^	43.81^a^^b^	0.43	0.042	0.348
**Back Fat Thickness(%)**	0.49^a^	0.45^a^^b^	0.39^b^	0.01	0.006	0.692
**Rib eye area (%)**^b^	13.49^a^	11.61^b^	10.21^b^	0.54	0.004	0.220
**Warm CW**^**c**^**(%)**	11.87	11.24	11.28	0.22	0.225	0.650
**Cold CW (%)**	11.71	11.04	11.16	0.22	0.259	0.754
**Meat (%)**	62.20	59.31	61.65	0.52	0.144	0.861
**Bone (%)**	29.23	31.45	29.50	0.57	0.199	0.844
**SCFAT**^**d**^**(%)**	2.82	2.73	3.07	0.13	0.492	0.951
**IMFAT**^**e**^**(%)**	5.75	6.51	5.78	0.25	0.693	0.431
**Primal cuts** **(%)**						
** Neck**	6.59	6.72	6.57	1.14	0.212	0.254
** Shoulder**	19.81	18.78	19.39	2.66	0.770	0.737
** Breast**	33.08	33.83	32.95	2.28	0.534	0.655
** Loin**	15.14	15.62	16.38	2.08	0.449	0.899
** Leg**	25.39	25.05	24.72	1.99	0.120	0.831

^a^LR: low *n*-6:*n*-3 PUFA ratio (2.27:1); MR: medium *n*-6:*n*-3 PUFA ratio (5.01:1); HR: high *n*-6:*n*-3 PUFA ratio (10.38:1).

^b^Rib eye area (REA) and back fat thickness were measured at locations between 12^th^-13^th^ ribs.

^c^CW: cooking weight

^d^SCFAT: subcutaneous fat.

^e^IMFAT: intermuscular fat. In general, the different levels of *n*-6:*n*-3PUFA ratios in the diet had no significant effect on the primal cut composition. Means with different superscripts within a row indicate a *P*-value ≤0.05

### Meat quality

The color of the meat aged (1, 3 and 6 days) of the LD muscle are presented in Table 5. The different levels of dietary *n*-6:*n*-3 PUFA ratios in post-mortem period had effects on L* (lightness), a* value (redness), b* values (yellowness), Chroma and Hue angle of the LD muscle. The LD muscle color was lighter (higher L* value; *P*<0.05) after 6 days of the postmortem period than other treatments at day 1 and 3 of the post-mortem periods. Increasing post-mortem days reduced a* and b* in all treatments. The different dietary *n*-6:*n*-3 PUFA ratios did not change (*P*>0.05) the a* and b* values at the same days of the post-mortem period. The LR group significantly decreased L* in LD muscle after 1 day and with increasing post-mortem period as compared to the HR group. The Chroma or saturation values significantly (*P*<0.05) decreased with increasing post-mortem periods in all treatment groups (Table 6). The different dietary *n*-6:*n*-3 PUFA ratios did not affect the Chroma value at the same post- mortem time. The hue angle did not show significant differences (*P*>0.05) by the post-mortem or dietary treatment for all treatment groups.

**Table 5:** Effect of dietary *n*-6:*n*-3 PUFA ratios on meat color of *longissimus dorsi* during the post-mortem period in goats.

Items	Treatments	Post-mortem periods			SEM
		1day	3day	6day	
**Lightness(L**^1^**)**	LR	34.83^b^^y^	38.68^ab^^x^	39.40^b^^x^	0.54
	MR	33.13^b^^y^	36.95^b^^x^	37.49^b^^x^	0.48
	HR	37.89^a^^y^	40.58^a^^x^	41.16^a^^x^	0.68
**Redness (a**^2^**)**	LR	10.68^x^	9.57^y^	8.85^y^	0.19
	MR	10.27^x^	9.01^y^	8.10^y^	0.26
	HR	10.11^x^	8.60^y^	7.64^y^	0.24
**Yellowness (b**^3^**)**	LR	10.60^x^	10.35^x^	9.04^x^	0.33
	MR	10.12^x^	9.00^xy^	8.10^y^	0.27
	HR	10.08^x^	9.13^y^	8.75^y^	0.2
**b**^3^**/a**^2^	LR	0.98	1.11	1.03^ab^	0.04
	MR	0.99	1.01	1.02^ab^	0.03
	HR	1.01	1.09	1.14^a^	0.03
**Hue angle (Hue)**	LR	43.93	46.41	45.39	1.06
	MR	44.67	44.62	45.15	0.85
	HR	44.81	46.9	47.92	0.84
**Chroma (C**^4^**)**	LR	15.00^x^	14.28^x^	12.71^y^	0.27
	MR	14.44^x^	12.80^y^	11.48^y^	0.32
	HR	14.32^x^	12.60^y^	11.74^y^	0.26

LR: low *n*-6:*n*-3 PUFA ratio (2.27:1); MR: medium *n*-6:*n*-3 PUFA ratio (5.01:1); HR: high *n*-6:*n*-3 PUFA ratio (10.38:1).

^x and y^ denote significant differences between days of the post-mortem period (*P*<0.05).

^a,b^denote significant differences within days of the post-mortem period (*P*<0.05). L^1^, Measure of darkness to lightness (a greater value indicates a lighter color). a^2^, Greater value indicates redder color. b^3^, Greater value indicates more yellow color. C^4^, Chroma or saturation index is a measure of total color/vividness of color (greater value indicates greater total color/more vivid color) C^4^ = √a2*b2. Hue, Hue angle = tan−1 (b/a)*180/π. L = linear and Q = quadratic effects. Post-mortem periods significantly (*P*<0.05) increased L* value in LD muscle of goats fed with all treatment groups after 7 days post-mortem as compared to 1 day of aging.

**Table 6:** Effect of dietary *n*-6:*n*-3 PUFA ratios on meat pH, drip loss (%), cooking loss (%) and shear force (kg) of *Longissimus dorsi* in goats.

Items	Treatments^2^	Post-mortem periods			SEM^3^
		1day	3day	6day	
**pH**	LR	6.62	6.84	6.81	0.03
	MR	6.76	6.99	6.84	0.03
	HR	6.56	6.7	6.76	0.01
**Drip loss**	LR	1.86^a^^y^	2.30^a^^xy^	2.63^x^	0.08
	MR	1.66^ab^^z^	2.03^b^^y^	2.79^x^	0.05
	HR	1.62^b^^z^	2.22^ab^^y^	3.18^x^	0.1
**Cooking loss**	LR	31.99^y^	35.65^y^	41.59^x^	0.74
	MR	29.24^y^	33.52^y^	38.85^x^	0.39
	HR	30.51^z^	35.37^y^	41.39^x^	0.38
**WBSF**^**1**^	LR	2.83^b^^x^	2.54^xy^	2.35^b^^y^	0.08
	MR	3.04^ab^^x^	2.61^xy^	2.45^a^^y^	0.09
	HR	3.14^a^^x^	2.66^y^	2.55^a^^y^	0.07

^1^WBSF: Warner–Bratzler shear force

^2^Treatments = LR: low *n*-6:*n*-3PUFA ratio (2.27:1); MR: medium *n*-6:*n*-3PUFA ratio (5.01:1); HR: high *n*-6:*n*-3PUFA ratio (10.38:1).

^3^SEM: standard error of mean.

^x,y and z^ denote significant difference (*P*<0.05) between days of post-mortem

^a and b^denote significant difference (*P*<0.05)within day of post-mortem.

The pH of meat samples (Table 6) at 1, 3 and 6 days post-mortem was similar for all groups (*P*>0.05). The percentages of drip loss in the LD muscle at different post-mortem periods were significantly different (*P*<0.05). Increasing post-mortem periods increased significantly (*P*<0.05) the percent (%) of drip loss (Table 6) in all treatment groups. The percentage of cooking loss (Table 6) in the LD muscle of goats fed different *n*-6:*n*-3 PUFA ratios was significantly (*P*<0.05) different at the same aging period. The 6-day post-mortem periods significantly increased (*P*<0.05) the cooking loss of all dietary treatments compared to 1-day aging period.

The Warner–Bratzler shear force (WBSF) of the LD muscle was significantly affected (*P*<0.05) across the different dietary *n*-6:*n*-3PUFA ratios at different post-mortem periods as shown in Table 6.The highest WBSF value belonged to the HR among of all treatments at day 1 post-mortem but it was not significantly different. The post-mortem was more effective in reducing the WBSF value (*P*<0.05) in the LD muscle. The aging significantly decreased the WBSF value in all treatment groups.

**Fig 1:**
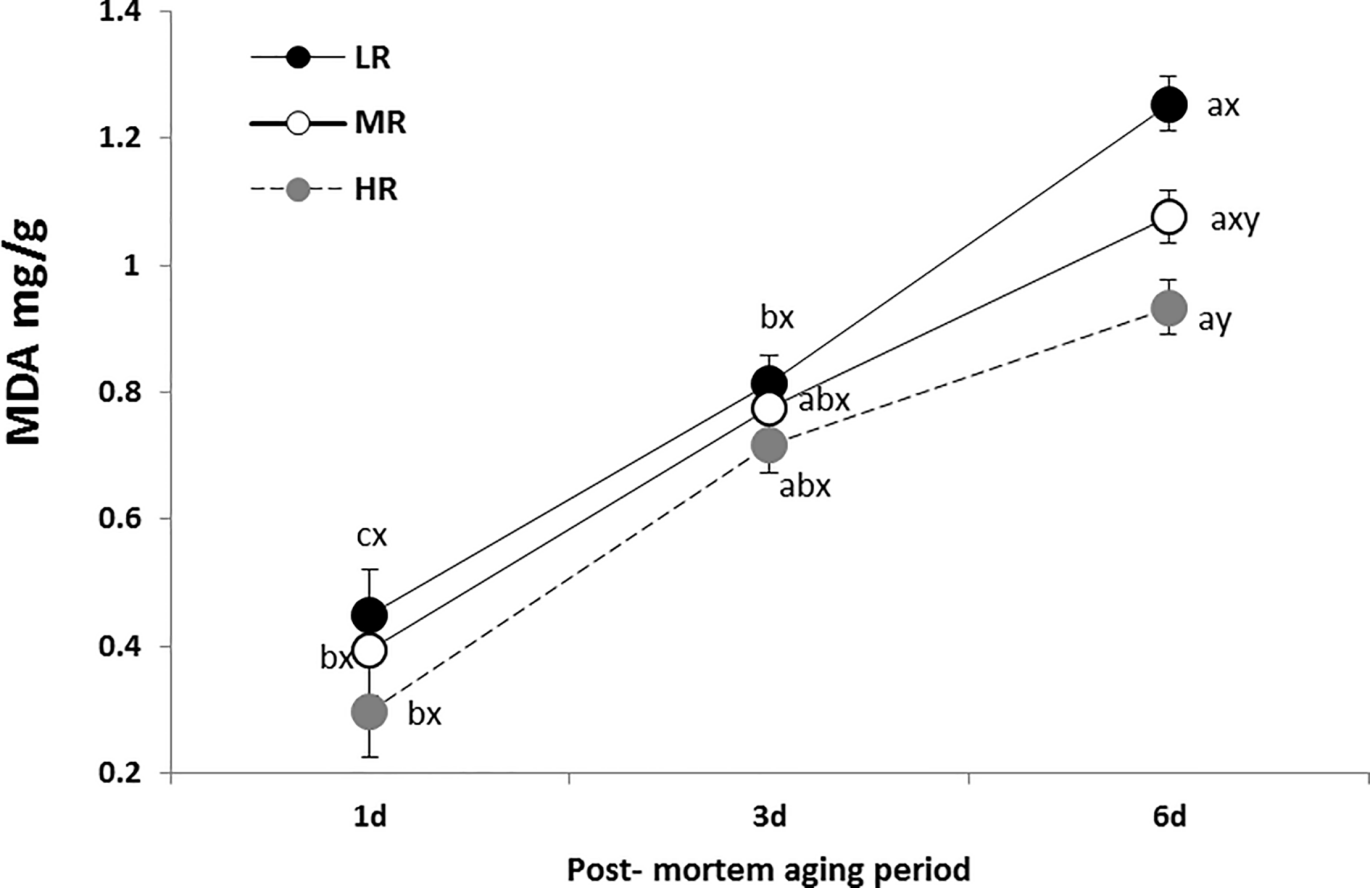
Effect of dietary *n*-6:*n*-3 PUFA ratios on lipid oxidation of *longissimus dorsi* in goats. LR: low *n*-6:*n*-3 PUFA ratio (2.27:1); MR: medium *n*-6:*n*-3 PUFA ratio (5.01:1); HR: high *n*-6:*n*-3 PUFA ratio (10.38:1). Vertical bars are standard error. ^x and y^ denote significant difference within day of post-mortem; ^a, b and c^ denote significant difference between days of post-mortem.

The TBARS values for the LD muscles across all the dietary *n*-6:*n*-3 PUFA ratios after a 6 day post-mortem period were significantly (*P*<0.05) higher than that on day 1 (Fig 1). The highest TBARS value belonged to the LR group compared to the HR treatment group after 6-days of aging. In the HR treatment, the rate of lipid oxidation was not significantly (*P*>0.05) lower compared to other treatments at the different post-mortem period. The aging period significantly (*P*<0.05) increased the TBARS value in all treatment groups (Fig 1).

### Effect of dietary *n*-6:*n*-3 PUFA ratios on FA content of experimental diets and *longissimus dorsi* tissue

The FA composition of the LD muscle of goats fed different dietary *n*-6:*n*-3 PUFA ratios is presented in Table 7. Among all FA contents, C18:1*n*-9 content in the LD muscle was the highest, ranging from 41.94 to 41.45% of identified FAs, followed by C16:0 (18.65–17.96% of identified FAs) and C18:0 (15.74–14.80% of identified FAs).The various dietary ratios of *n-6*:*n-3* PUFA did not significantly affect (*P*>0.05) the concentrations of C10:0, C12:0, C14:0, C15:0, C15:1, C16:0, C16:1n-7, C17:0, and C18:1n-9 in the LD muscle. The proportion of muscle FA having 18 carbons was quite consistent across the three treatment groups, averaging between 66.66 to 67.42%. Results also revealed that increasing dietary ratios of dietary *n*-6:*n*-3 PUFA resulted in a linear decrease in the concentrations of C18:0 (*P* = 0.03), cis-12 trans-10 CLA (*P* = 0.05), C18:3*n*-3, C20:5*n*-3, C22:5*n*-3, C22:6*n*-3 (*P* = 0.001), and a linear increase in the concentrations of C17:1, C18:1 trans-11 (*P* = 0.01), cis-9, trans-11 CLA, C18:2*n*-6 (*P* = 0.01) and C20:4*n*-6 (*P* = 0.001) in the LD muscle. The concentration of C10:0 (*P* = 0.05) increased quadratic as dietary *n*-6:*n*-3 PUFA ratios increased. The total SFA, UFA and MUFA were not affected (*P*>0.05) by the dietary *n*-6:*n*-3 PUFA ratios. However total *n-*3 PUFA decreased linearly with increasing *n*-6:*n*-3 PUFA ratios. In contrast, the sum of CLA isomers showed a positive linear increase to increasing *n*-6:*n*-3 PUFA ratios. The major increase in this ratio was observed in the HR diet with the highest level of *n*-6:*n*-3 PUFA ratios.

**Table 7:** Effect of dietary *n*-6:*n*-3 PUFA ratios on FA composition (% of identified FAs) of *longissimus dorsi* in goats.

Fatty Acids	Dietary *n*-6:*n*-3 PUFA ratios^1^			SEM	*P-*value	
	LR	MR	HR		Linear	Quadratic
C10:0, Capric	0.61^b^	0.95^a^	0.68^b^	0.04	0.643	0.050
C12:0, Lauruic	2.22	2.45	2.51	0.09	0.638	0.342
C14:0, Myristic	2.41	2.83	2.42	0.13	0.595	0.387
C14:1, Myristoleic	0.17^b^	0.26^a^	0.24^a^	0.02	0.003	0.818
C15:0, Pentadecanoic	1.65	1.25	1.23	0.10	0.338	0.278
C15:1, Pentadecenoic	0.15	0.14	0.13	0.01	0.250	0.196
C16:0, Palmitic	18.65	18.12	17.96	0.18	0.104	0.644
C16:1n-7, Palmitoleic	1.83	2.00	2.07	0.06	0.308	0.451
C17:0, Margaric	0.69	0.81	0.84	0.03	0.105	0.596
C17:1, Margaroleic	0.49^c^	0.55^b^	0.62^a^	0.04	0.005	0.339
C18:0, Stearic	15.74^b^	15.63^b^	14.80^a^	0.17	0.031	0.738
C18:1*n*-9, Oleic	41.94	41.52	41.45	0.43	0.272	0.191
C18:1, trans-11 Vaccenic	1.11^c^	1.38^b^	2.27^a^	0.05	0.012	0.582
C18:2*n*-6, Linoleic	6.17^c^	6.46^b^	7.30^a^	0.20	0.014	0.860
CLA cis-9, trans-11	0.71^c^	0.75^b^	0.96^a^	0.04	0.001	0.131
CLA cis-12 trans-10	0.34^a^	0.33^ab^	0.32^b^	0.02	0.053	0.399
C18:3*n*-3, α-Linolenic	1.09^a^	0.58^b^	0.32^c^	0.07	0.001	0.101
C20:4*n*-6, Arachidonic	2.79^c^	3.16^b^	3.32^a^	0.07	0.001	0.799
C20:5*n*-3, Eicosapentaenoic	0.57^a^	0.35^b^	0.22^c^	0.03	0.001	0.115
C22:5*n*-3, Docosapentaenoic	0.61^a^	0.44^b^	0.30^c^	0.03	0.001	0.067
C22:6*n*-3, Docosahexaenoic	0.06^a^	0.03^b^	0.03^b^	0.001	0.001	0.147
SFA^2^	41.96	42.04	40.43	0.30	0.542	0.626
UFA^3^	58.04	57.96	59.57	0.30	0.311	0.626
MUFA^4^	45.69	45.85	46.78	0.44	0.183	0.222
PUFA *n*-3^5^	2.33^a^	1.40^b^	0.88^c^	0.12	0.001	0.101
PUFA *n*-6^6^	8.96	9.62	10.63	0.19	0.087	0.210
Total trans FA^7^	1.11^c^	1.38^b^	2.27^a^	0.05	0.012	0.582
Total CLA^8^	1.05^b^	1.09^b^	1.28^a^	0.05	0.001	0.200
*n*-6:*n*-3 ratio^9^	3.84^c^	6.88^b^	12.09^a^	0.68	0.001	0.520
UFA:SFA	1.38	1.38	1.47	0.02	0.568	0.632
PUFA:SFA ratio	0.27	0.26	0.28	0.01	0.471	0.061

^1^LR: low *n*-6:*n*-3 PUFA ratios; MR: medium *n*-6:*n*-3 PUFA ratios; HR: high *n*-6:*n*-3 PUFA ratios.

^2^SFA = sum of C10:0 +C12:0 +C14:0+ C15:0+ C16:0+ C17:0+ C18:0.

^3^UFA = sum of C14:1+C16:1+C17:1+C18:1+C18:2+C18:3+C20:4,C22:6,C20:5*n*-3+C22:5–3+C22:6*n*-3.

^4^MUFA = sum of C14:1+C16:1+C17:1+C18:1.

^5^PUFA *n*-3 = sum of C18:3*n*-3+C20:5*n*-3+C22:5 *n*-3+C22:6*n*-3.

^6^PUFA*n*-6 = sum of 18:2*n*-6+20:4*n*-6.

^7^Total trans FA = C18:1trans

^8^Total CLA = sum of cis-9, trans-11CLA + cis-12 trans-10CLA

^9^*n*-6:*n*-3 PUFA ratios = (C18:2*n*-6 + C20:4n-6) / (C18:3*n*-3 + C20:5*n*-3 + C22:5*n*-3 + C2). Means with different superscripts ^(a,b,c)^ within a row indicate—significant difference at *P* ≤0.05.

### Expression of PPARα, PPARγ and SCD genes in *longissimus dorsi* muscle

**Fig 2:**
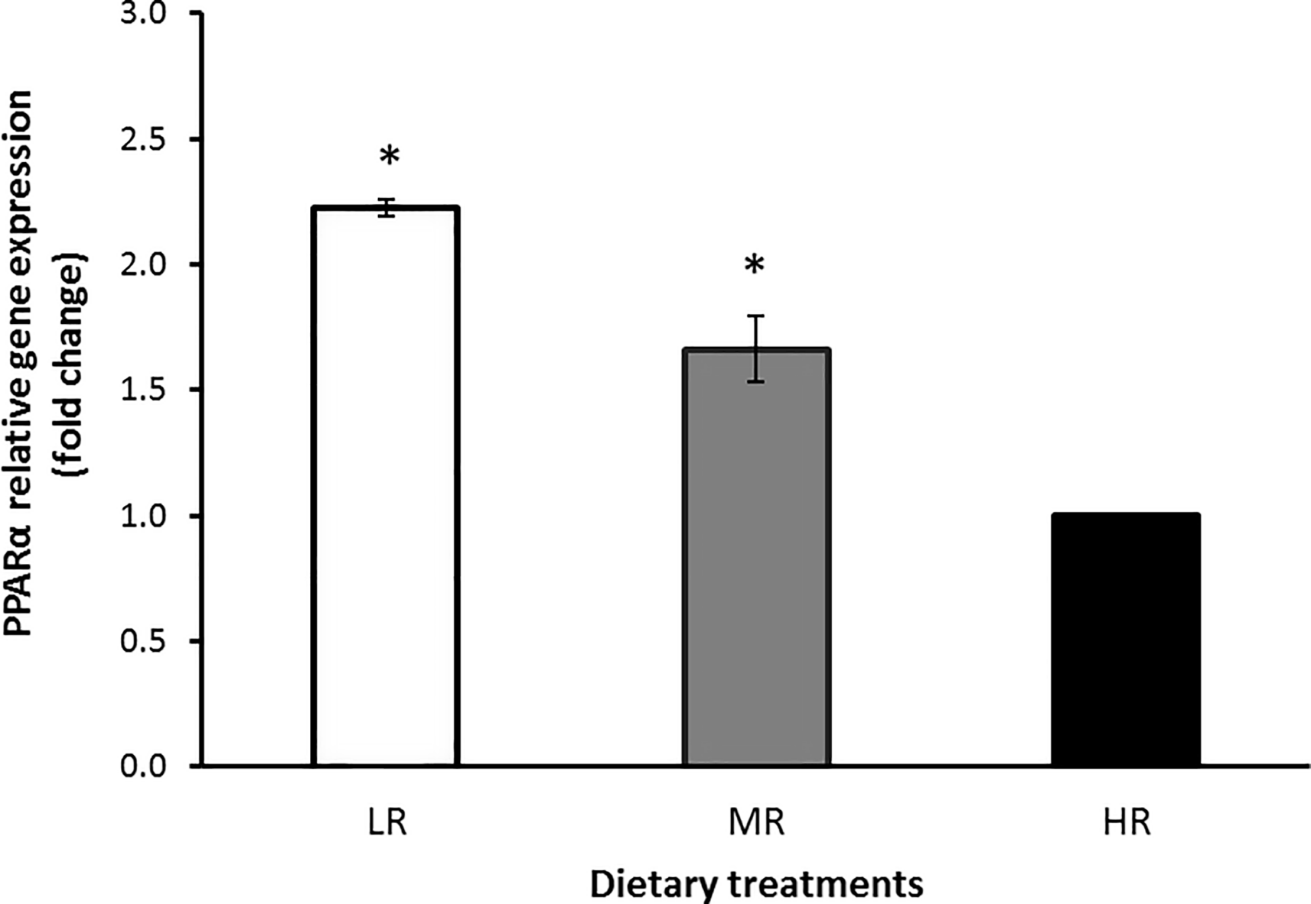
Expression of PPARα in the LD muscle of treatment groups compared to HR group. Values were normalized with a housekeeping gene, β-actin. Then, treated samples were expressed relative to gene expression of HR group. Values are means ± 1 standard error bar. Values indicated by * show significant difference compared with HR group (*P*< 0.05). HR: high *n*-6:*n*-3 PUFA ratio (10.38:1), MR: medium *n*-6:*n*-3 PUFA ratio (5.01:1) and LR: low *n*-6:*n*-3 PUFA ratio (2.27:1).

**Fig 3:**
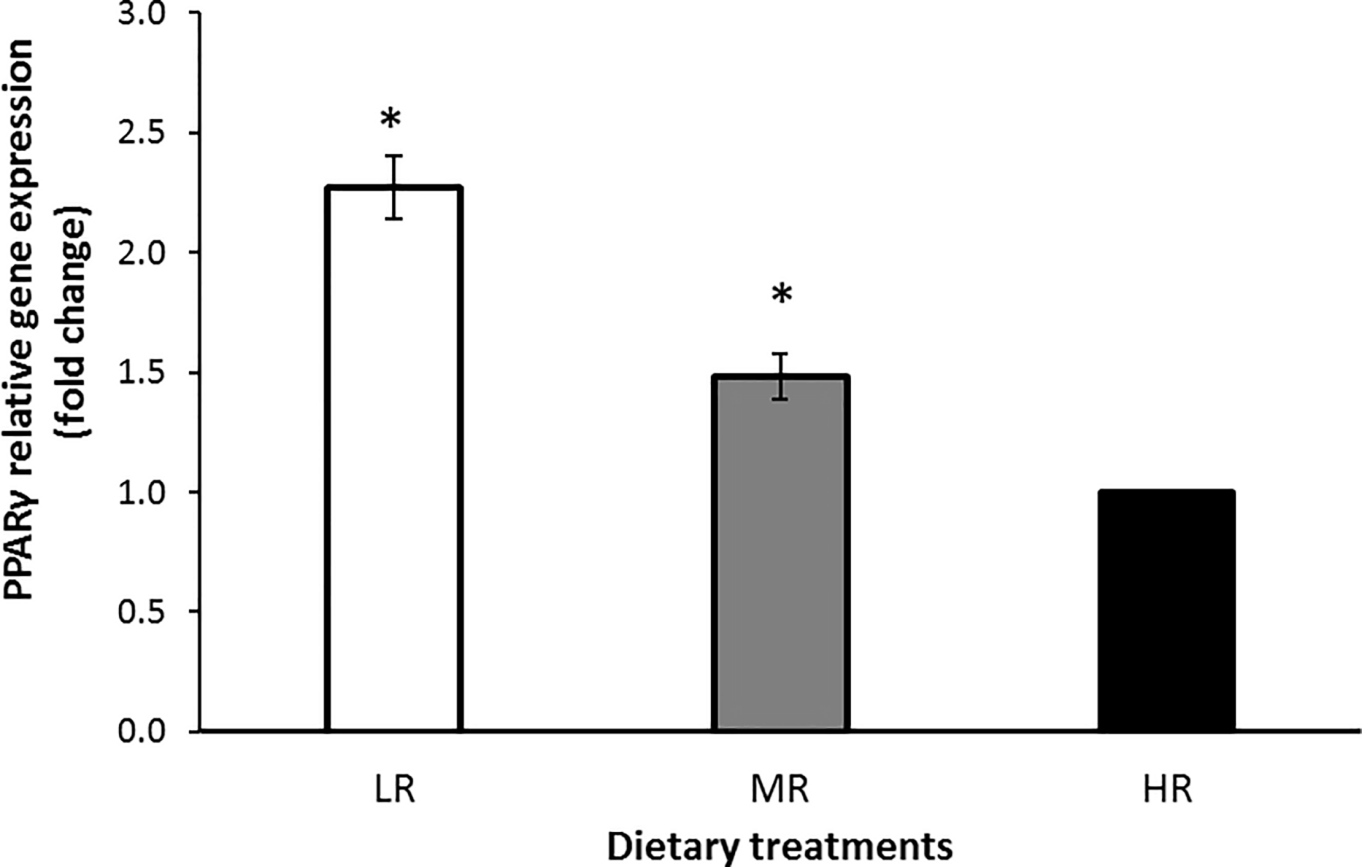
Expression of PPARγ in the LD muscle of treatment groups compared to HR group. Values were normalized with a housekeeping gene, β-actin. Then, treated samples were expressed relative to gene expression of HR group. Values are means ± 1 standard error bar. Values indicated by * show significant difference compared with HR group (P<0.05). HR: high *n*-6:*n*-3 PUFA ratio (10.38:1), MR: medium *n*-6:*n*-3 PUFA ratio (5.01:1) and LR: low *n*-6:*n*-3 PUFA ratio (2.27:1).

**Fig 4:**
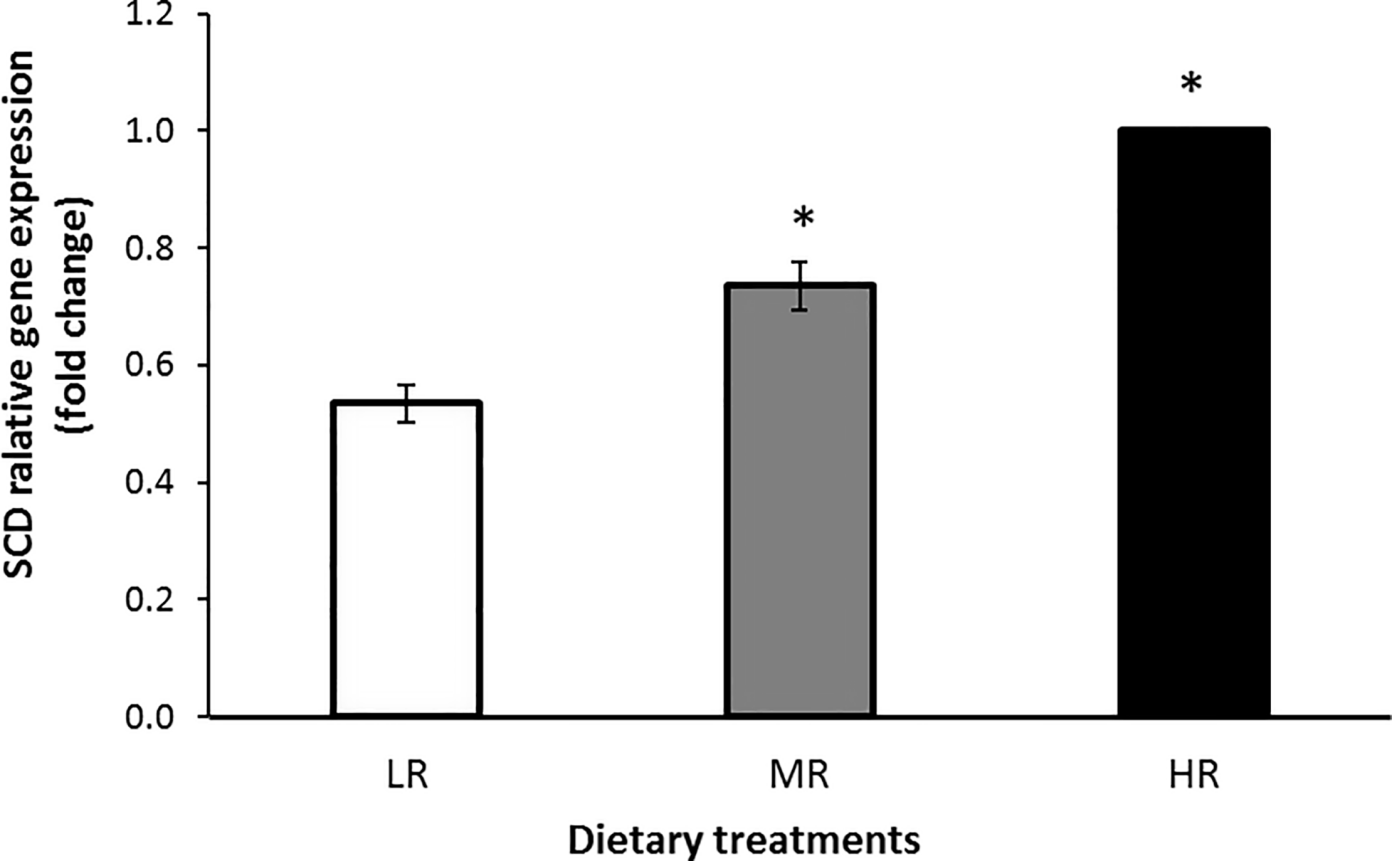
Expression of SCD in the LD muscle of treatment groups compared to HR group. Values were normalized with a housekeeping gene, β-actin. Then, treated samples were expressed relative to gene expression of HR group. Values are means ± 1 standard error bar. Values indicated by * show significant difference compared with the HR group (*P*< 0.05). HR: high *n*-6:*n*-3 PUFA ratio (10.38:1), MR: medium *n*-6:*n*-3 PUFA ratio (5.01:1) and LR: low *n-*6:*n*-3 PUFA ratio (2.27).

Figs 2, 3 and 4 show the relative gene expression in the LD muscle of goats fed with varying dietary *n*-6:*n*-3 PUFA ratios. The PPARα gene showed a higher level of expression in the LR group compared with the HR group (*P*<0.05), indicating that lowering dietary *n*-6:*n*-3PUFA ratios had upregulated the PPARα gene expression. The PPARγ gene was also higher in the LR groups (*P*<0.05) compared with the MR group suggesting that the expression of the PPARγ gene was also upregulated in the muscle of the LR group compared to HR animals. The SCD gene expression showed a significant (*P*<0.05) reduction in the LR group compared to the HR group suggesting that the SCD gene was down-regulatedby the LR treatment.

## Discussion

Although there are some reports describing the manipulation of FA composition in animal meat [26, 27, 28], little research has been done to study the effects of different dietary *n*-6:*n*-3 PUFA ratios on meat quality, tissue FA profiles, and the expression of lipogenic genes. The high intake of *n*-6 PUFA has the potential to alter the FA profile of the membrane phospholipids with higher proportions of LA and arachidonic acids, which in turn might change the quality of meat, carcass characteristics, and also expression of lipogenic genes in animals [29]. We demonstrated that the optimal *n*-6:*n*-3 PUFA ratio of 2.27:1 exerted beneficial effects on meat FA profiles, leading towards an enrichment in n-3 PUFA and conjugated LA in goat intramuscular fat. Our data showed that there were no effects on the growth performance, most of the carcass characteristics, and the primal cuts of goats when they were fed with different *n*-6:*n*-3 ratio diets, which can be explained by either the same amount of fat was in the diets or similar feed intake. The intake of experimental feed averaging 3.7% of the BW daily indicated that the goat consumed all the feed offered. Similar to the current results, Kim et al [30] found no effects of dietary*n*-6:*n*-3 PUFA ratios on BW, dry matter intake, and ADG of lambs.

Our findings showed that there were no effects on meat color of goats when they were fed with different *n*-6:*n*-3 ratio diets, but the color values in all treatment diets were changed during the aging period. The results of this study are consistent with those of Juarez et al [31], indicated no significant effects of dietary flaxseed and (or) α-tocopheryl acetate supplements on L*, a* or b* values of pig, but the aging period showed a significant change in the color of the pig. The color of meat in ruminants depends on the animal age, weight, exercise, and meat pH, and were similar across all three treatment groups. These results were expected since their pre-slaughter energy, consumption was similar and the goats were managed similarly pre-slaughter hence no effect on the muscle color or muscle pH was anticipated and all data recorded can be considered “normal”.

Saturation values (purity of red color) which decreased over the 6 days of aging period, changing from red to brown, commonly observed in displaying meat, were in agreement with Karami et al [32]. In this study, there was a tendency towards a significantly different effect by the aging period. Moreover, if the * values for day 1 and day 6 were analyzed separately, there would be a significant treatment × day interaction for day 1 meat. This is due to a* values for muscles from the LR dietary treatments being higher than the other treatments. The Warner–Bratzler shear force was not affected by the dietary *n*-6:*n*-3 PUFA ratios in the LD muscle, similar to the report by Lee et al [33] who found that the shear force values of chevon chops after 24 h post-mortem were not influenced by the dietary post weaning diets. The post-mortem periods significantly improved tenderness at 6 days, particularly in the LR treatments in this study. One possible explanation is the location of neutral lipids in the fat cells within the perimysium which could have a physical effect in separating muscle fiber bundles, beginning the process of tenderization of ‘‘opening up the muscle structure” [34].

The composition of FA determines the firmness/oiliness of adipose tissue and the oxidative stability of muscle, which in turn affects flavor and muscle color [35]. Malondialdehyde as measured by the TBARS analysis is formed primarily by the oxidation of UFA, with the reaction being more intense as the level of unsaturation of the fat increases [36]. The non-significant difference from the TBARS value between treatment groups at the same aging time in the present study can be explained by the occurrence of similar amounts of total PUFA in the LD muscle. The aging period significantly increased the TBARS value in all treatment groups, which are in agreement with other previous studies [37] and [38].

In this study, the lack of differences between the treatment groups for the pH of warm and cold carcass could be due to the fact that the different dietary *n-*6:*n-*3 PUFA ratios had no effects on the glycogen content of the meat at slaughter. Glycogen plays an important role in the pH value of meat [39]; however, the percentage of glycogen in the meat was not determined in the current study, which necessitates more studies in future.

Generally, a positive relationship has been reported between the concentrations of dietary LA and tissue cis-9, trans-11 CLA in grazing heifer tissues fed diets supplemented with plant oil-enriched concentrates [40]. The C18:1 *trans*-11 isomer is an intermediate product in the microbial BH of dietary C18:1n-9, LA, and LNA [5]. There is a positive relationship between C18:1 trans-11 and cis-9, trans-11CLA in intramuscular lipid [40]. In this study, higher C18:1 trans-11 and cis-9, trans-11 CLA concentrations in LD muscle were observed in HR group. The C18:1 trans-11 concentration in LD muscle, which could be converted to cis-9, trans-11 CLA by the muscle, increased linearly with increasing dietary *n*-6: *n*-3 PUFA ratios. This result can be explained by the increase in LA levels in goats fed the HR diet, indicating that more LA was isomerized to *cis*-9, *trans*-11 CLA and hydrogenated to C18:1 *trans*-11 in the rumen of goats fed the higher *n*-6:*n*-3 PUFA ratio leading to more deposition in the muscle. Jeronimo et al [41] reported that the concentrations of C18:1 *trans*-11 as well as *cis*-9, *trans*-11 CLA in *longissimus dosri* muscle increased in lambs fed diets with high *n-*6 were then those fed diets with high *n*-3. Therefore, the synthesis of these isomers from C18:2n-6 may have been more efficient than that from C18:3*n*-3. Supplementation of SO would therefore result in more rumen-derived *cis*-9, *trans*-11 CLA and less diversity of BH-derived FA than with linseed oil. The content of *cis*-9, *trans*-11 CLA in meat increased with increasing the *n*-6:*n*-3 PUFA ratio, confirming the previous results obtained by [30]. In addition, Noci et al [42] observed that the *cis*-9, *trans*-11 CLA content in *longissimus* muscle was higher in heifers supplemented with sunflower oil than with linseed oil. The explanation might be found in ruminal BH pathways of C18:2*n*-6 and C18:3*n*-3. Most of the cis-9, trans-11 CLA present in tissues derives from endogenous desaturation of C18:1 *trans*-11 which originates during BH of both C18:2n-6 and LNA [43]. However, *cis*-9, *trans*-11 CLA is also synthesized by direct isomerization of LA in the rumen.

A greater escape of LA than that of LNA from the rumen in the present study would result in lower concentrations of C18:0 in muscle. Jeronimo et al [41] reported that the concentration of LA in LD muscle of lambs could be increased dramatically from 5.09 to 9.36 (%) of identified FA when the dietary *n-*6:*n*-3 PUFA ratio was changed from 9.06 to 0.38 (%). Lower concentrations of stearic acid in muscle would likely result in lower concentrations of C18:1n-9 because C18:0 in muscle serves as a substrate for the synthesis of C18:1n-9, by the Δ-9 desaturase enzyme [42]. The LD muscle increased the concentrations of C18:1 trans-11.

The concentration of LNA was greatest in LD muscle (1.09%) of goats fed the LR diet. The results of this study are consistent with those of Karami et al [37] who reported that the addition of 3% of canola oil (source of LNA) in the diet of goat kids increased the LNA concentration in LD muscle after 84 days of animal trial. The long-chain *n*-3 PUFA(C20:5*n*-3, C22:5*n*-3 and C22:6*n*-3) in LD muscle are the metabolic products of LNA. The concentrations of C20:5*n*-3, C22:5*n*-3 and C22:6*n*-3 followed a similar linear response to LNA concentrations in this study. Jeronimo et al [41] documented that the concentration of C20:5n-3, C22:5n-3 and C22:6n-3 were increased in ruminant muscle when they were fed high levels of LO rather than SO. The capacity of conversion of LNA to health promoting *n*-3 LC-PUFA is limited in humans, which strengthens the importance of its dietary supply [44]. The probable mechanism can be the competition between LA and LNA for desaturation and elongation enzymes, which affect the conversion to long chain FA derivatives [45]. The supplementation of ruminant diets with fish oil has been shown to be more effective to increase the EPA and DHA in meat than lipid sources rich in LNA [46]. However, using fish oil may decrease the meat shelf life and affect the flavor [47] as well as on the sustainability of increased use of fish oil in the food chain [48]. The HR treatment showed less *n*-3 LC-PUFA rather than the LR treatment. Therefore, these results showed that the lowering the *n*-6:*n*-3 PUFA ratio may be one possible alternative to increase the *n*-3 LC-PUFA in goat meat.

Decreasing the dietary *n*-6:*n*-3 PUFA ratio by 4.58 fold (from 10.38 to 2.27) via oil supplementation changed that same ratio by 3.15 fold in the LD muscle from 12.09 to 3.84. When high concentrate diets are fed, replacing oil supplement having a high proportion of LA with one having a high proportion of LNA, such as LO, can reduce by half the *n*-6:*n*-3 PUFA ratio in muscle, thus making it a more attractive meat for hyper-cholesterolemic or diabetic people. Others have shown an increase in the *n*-6:*n*-3 PUFA ratios in LD muscle when feeding a fat source having a high concentration of LA, namely soybean oil. When soybean oil (0 or 8% of dietary DM) was fed to lambs, the *n-*6:*n*-3 PUFA ratio in *longissmus thorax* increased from 2.1 to 5.0 [49]. The *n*-6:*n*-3 PUFA ratio in food should range between 1 and 4 [50] and the *n*-6: *n*-3 PUFA ratio found in the LR treatment (3.84) was within this range. In the LR treatment, only 53.15% of *n*-3 PUFA were *n*-3 LC-PUFA. This can be important considering that health benefits of the *n*-3 FA are mostly associated with *n*-3 LC-PUFA and that metabolism of LNA in humans is limited [44].

In this study, it was found that the mRNA expression of genes involved in the lipid metabolism of goats was altered by changes in dietary *n*-6:*n*-3 ratios. Specifically, the mRNA expression of PPARα and PPARγ were up-regulated in the LD muscle. The PPAR-α responds to changes in the dietary fat by activating the expression of various enzymes involved in fatty acyl-CoA formation and hydrolysis, FA elongation and desaturation, and FA oxidation [51]. We speculated that as PPARγ is related to the expression regulation of several genes encoding proteins involved in adipocyte metabolism, and it could also be a candidate gene affecting the fat deposition, including intramuscular fat deposition [52]. The lowering of the dietary *n*-6:*n*-3 PUFA ratio might stimulate PPAR target gene expressions such as lipoprotein lipase (LPL), FA transport protein (FATP) and acyl-CoA synthase (ACS) [53, 54].

Previous studies have revealed that the PUFA activated PPAR efficiently despite the fact that very long chain FAs and short-chain FAs (<Cl0) cannot activate the PPARα and PPARγ because these FAs are exclusively metabolized in the peroxisomes [55]. Al-Hasani and Joost [51] also showed that lowering the *n*-6:*n*-3 PUFA ratio in the rodent diet can increase PPARγ activity in target tissues which are associated with increased insulin sensitivity. An alternative explanation is that the effects of FAs on PPARγ signaling are mediated through changes in PPARγ activity rather than changes in gene expression, which was not measured in this study, since FAs have been shown to act as natural ligands of the PPARγ gene in other studies [56].

In this study, the expression levels of SCD gene in the LD muscle of goats fed the diets with n-6:n-3 PUFA ratios of 2.27:1 (LR) compared to MR and HR were markedly reduced. Stearoyl-CoA desaturase (SCD) is a key enzyme in FA metabolism by modifying the ratio of MUFAs to SFAs, which can affect lipoprotein metabolism and adiposity [57].The expression of SCD is known to be strongly modulated by several nutrients such as FAs, carbohydrates, hormones [23, 58], and cholesterol [59].Waters et al [23] found a negative relationship between SCD gene expression and *n*-3 PUFA, EPA (C20:5*n*-3), DHA (C22:6*n*-3) and LNA in beef cattle which is in agreement with the present finding where the downregulation of SCD occurred in the LR treatment group with the highest LNA level. Belinger et al [60] showed that feeding a mixture of *n*-3 PUFA, EPA, DHA, and LNA resulted in a 50% suppression of SCD mRNA in the rat liver. Nutrients, especially FAs, have been shown to regulate SCD at both enzyme activities [61] and transcriptional level [62]. The results of the current study have important implications with regard to ingesting *n*-3 PUFA, which may have negative effects on the *de novo* synthesis of CLA in the human muscle [63, 64] through potential reductions in the SCD gene expression as shown in this study. However, because a positive relationship of n-6 PUFA, and particularly the *n*-6:*n*-3 PUFA ratios, with SCD gene expression was observed, the correct balance of dietary *n*-6:*n*-3 PUFA ratio appears to be of critical importance to achieving optimal SCD gene expression levels and, in turn, CLA production in the muscle tissue [65, 66].

## Conclusion

In conclusion, we revealed the relationship between dietary PUFAs and tissue FA profiles and demonstrated that decreasing the dietary *n*-6:*n*-3 PUFA ratios increased the LNA and *n*-3 LC-PUFA concentrations in the goat meat, with the highest value of *n*-3 LC-PUFA achieved with lowest *n*-6:*n*-3 PUFA ratio (LR, 2.27: 1). These findings suggest that a balanced dietary *n*-6:*n*-3 PUFA ratio plays an important role in mRNA expression of lipogenic genes through the altered expression of PPAR𝛼, PPARγ, and SCD in the LD muscle and the utilization of lowest *n*-6:*n*-3 PUFA ratio is a valid approach for obtaining goat meat enriched with n-3 LC-PUFA.
