# Correction: Effects of dietary *n*-6: *n*-3 polyunsaturated fatty acid ratios on meat quality, carcass characteristics, tissue fatty acid profiles, and expression of lipogenic genes in growing goats

**DOI:** 10.1371/journal.pone.0222678

**Published:** 2019-09-12

**Authors:** Mahdi Ebrahimi, Mohamed Ali Rajion, Saeid Jafari, Mohammad Faseleh Jahromi, Ehsan Oskoueian, Awis Qurni Sazili, Yong Meng Goh, Morteza Hosseini Ghaffari

The second affiliation for the fifth author is not indicated. Ehsan Oskoueian is affiliated with: Mashhad Branch, Agricultural Biotechnology Research Institute of Iran (ABRII), Agricultural Research, Education, and Extension Organization (AREEO), Mashhad, Iran.
